# In Vitro Degradation of Mg-Doped ZrO_2_ Bioceramics at the Interface with Xerostom^®^ Saliva Substitute Gel

**DOI:** 10.3390/ma16072680

**Published:** 2023-03-28

**Authors:** Liliana Bizo, Marieta Mureşan-Pop, Réka Barabás, Lucian Barbu-Tudoran, Antonela Berar

**Affiliations:** 1 Department of Chemical Engineering, Faculty of Chemistry and Chemical Engineering, Babeş-Bolyai University, 11 Arany Janos Str., RO-400028 Cluj-Napoca, Romania; 2 Nanostructured Materials and Bio-Nano-Interfaces Center, Institute for Interdisciplinary Research on Bio-Nano-Sciences, Babeş-Bolyai University, 42 Treboniu Laurian Str., RO-400271 Cluj-Napoca, Romania; marieta.muresan@ubbcluj.ro; 3 Department of Chemistry and Chemical Engineering of Hungarian Line of Study, Faculty of Chemistry and Chemical Engineering, Babeş-Bolyai University, 11 Arany Janos Str., RO-400028 Cluj-Napoca, Romania; reka.barabas@ubbcluj.ro; 4 Electron Microscopy Center “Prof. C. Craciun”, Faculty of Biology and Geology, Babeș-Bolyai University, 5-7 Clinicilor Str., RO-400006 Cluj-Napoca, Romania; lucian.barbu@ubbcluj.ro; 5 Electron Microscopy Integrated Laboratory, National Institute for R&D of Isotopic and Molecular Technologies, 67-103 Donath Str., RO-400293 Cluj-Napoca, Romania; 6 Department of Prosthetic Dentistry, Faculty of Dental Medicine, Iuliu Haţieganu University of Medicine and Pharmacy, 32 Clinicilor Str., RO-400006 Cluj-Napoca, Romania; berar.antonela@umfcluj.ro

**Keywords:** bioceramics, magnesia-doped zirconia, XRPD, IR spectroscopy, SEM/EDS

## Abstract

Zirconia-based bioceramics, one of the most important materials used for dental applications, have been intensively studied in recent years due to their excellent mechanical resistance and chemical inertness in the mouth. In this work, the structural, morphological and dissolution properties of the Zr_1−x_Mg_x_O_2_ (x = 0.05, 0.1, 0.15, 0.2, 0.25, and 0.3) system, prepared by the conventional ceramic method, were evaluated before and after immersion in saliva substitute gel (Xerostom^®^, Biocosmetics Laboratories, Madrid, Spain), one of the most common topical dry mouth products used in dentistry. The X-ray powder diffraction (XRPD), Fourier transform infrared spectroscopy (FTIR) and scanning electron microscopy/energy-dispersive X-ray spectroscopy (SEM/EDS) techniques were employed to investigate the phase transformations and morphology of the ceramics during the degradation process in Xerostom^®^. In vitro analyses showed overall good stability in the Xerostom^®^ environment, except for the x = 0.05 composition, where significant t- to m-ZrO_2_ transformation occurred. In addition, the strong interconnection of the grains was maintained after immersion, which could allow a high mechanical strength of the ceramics to be obtained.

## 1. Introduction

Zirconia and zirconia-based ceramics are used for a wide range of clinical applications due to their improved material strength, enhanced aesthetic and high biocompatibility [1,2,3]. Besides many applications of these materials, medical and dental applications occupy an important position [4,5]. In dentistry, the main applications of zirconia include the fabrication of full and partial coverage crowns, veneers, fixed partial dentures, posts and/or cores, implant abutments or implants [6,7].

It is known that the mechanical properties and durability of zirconia ceramics are directly linked to their crystallography. In this direction, the evolution from one metastable polymorph (tetragonal phase, t-ZrO_2_) to the stable one (monoclinic phase, m-ZrO_2_) explains both the phase transformation toughening mechanism responsible for the high mechanical properties of zirconia and its sensitivity to low-temperature degradation (LTD) [8]. The stabilization of the t-ZrO_2_ phase at room temperature could be achieved by the addition of calcia (CaO), magnesia (MgO), ceria (CeO_2_) or yttria (Y_2_O_3_). The yttria-stabilized zirconia (YSZ) has proved to be a promising material due to its excellent properties and has been applied for years in various industries. It has been shown that YSZ can maintain its t-ZrO_2_ phase at room temperature and is considered the principal type of zirconia for current medical use [9]. Conversely, CaO- or MgO-stabilized zirconia systems, which are called PSZ (partially stabilized zirconia), are less expensive than YSZ. In addition, besides its low cost and availability, MgO is recognized as an effective antibacterial agent, which makes it an excellent candidate for biological applications. There are many nanoparticles with antimicrobial properties against a broad spectrum of microorganisms (ZnO, TiO_2_, silver nanoparticles, etc.), but many of them cause significant concerns regarding their toxicity due to the risks associated with heavy metal elements and their accumulation in the body. Conversely, magnesium oxide nanoparticles (MgO NP) represent an attractive alternative to ZnO and silver because MgO NP can be degraded and metabolized efficiently in the body. Moreover, the released degradation products of Mg^2+^ and OH^−^ ions can be effectively eliminated from the body if renal function is normal [10,11,12,13].

Zirconia’s interaction with the oral environment (fibroblasts, osteoblasts, dental pulp cells, macrophages), revealed good biocompatibility [14]. It was demonstrated that zirconia-based dental ceramics are chemically inert materials with no adverse effects on oral tissues and have been correlated with excellent cell adherence and no harmful systemic responses.

In recent years, many researchers have evaluated zirconia’s biocompatibility in vitro by monitoring different cell culture interactions with the biomaterial. The in vivo and in vitro experiments of Y-TZP (yttria-stabilized tetragonal zirconia polycrystal), the ceramic with superior mechanical properties compared to the conventional ones, show that it has good biocompatibility and no adverse reactions to cells and tissues [15].

Ichikawa et al. evaluated the in vivo tissue reaction and stability of PSZ ceramic by subcutaneous implantation for 12 months. The zirconia samples were completely encapsulated by a thin fibrous connective tissue, and they observed no changes of weight and three-point bending strength. Their result suggested that zirconia ceramic is biocompatible, and no degradation of zirconia ceramic occurred [16].

Sharanraj et al. evaluated the biocompatibility of a 3Y-TZP (3 mol% yttria-stabilized tetragonal zirconia polycrystal) specimen by in vitro analyses (direct contact test and agar diffusion method), using mouse fibroblast (L929 cell line). Their results confirmed that composition with t-ZrO_2_ phase revealed zero-grade cytotoxicity and the highest cell growth (93.17%) [17].

The research of Kazi et al. on 8Y-TZP (fully stabilized cubic phase zirconia with 8 mol% yttria), by cell adherence, cell proliferation analysis (using fibroblast-like cells L929) and cell differentiation analysis (using mouse bone marrow-derived mesenchymal stem cells—BMSC), demonstrated a suitable biological response. The results evidenced the biocompatibility of the cubic 8Y-TZP and suggest that yttrias with a higher zirconia content are not toxic to the cells, support a strong adhesion of cells on their surfaces, and promote cell proliferation and differentiation [18].

Wei et al. comparatively investigated zirconia and titanium in terms of the initial stem cell and preosteoblast cell adhesion and oxidative response. Human dental pulp stem cells (DPSC) and murine pre-osteoblasts (MC3T3-E1) cells were cultured on zirconia and titanium surfaces. The cell viability and morphology were monitored at 3, 12 and 24 h intervals. Their results show that zirconia revealed a relatively higher mean cell viability compared to titanium within 24 h culture, with significantly higher DPSC viability at 12 h after seeding (*p* < 0.05) [19].

The present study focuses on aspects related to the behavior of the Zr_1−x_Mg_x_O_2_ (x = 0.05, 0.1, 0.15, 0.2, 0.25 and 0.3) system in Xerostom^®^ saliva substitute gel, one of the most common topical dry mouth products used in dentistry. To the best of our knowledge, no literature data are available relating to Mg-doped zirconia behavior after immersion in Xerostom^®^. The materials were prepared by ceramic processing, which involves mixing, compaction and sintering. Moreover, to evaluate the stability of the prepared materials, the obtained bioceramics were structural and morphologically analyzed pre- and post-immersion by X-ray powder diffraction (XRPD), Fourier transform infrared spectroscopy (FTIR) and scanning electron microscopy/energy-dispersive X-ray spectroscopy (SEM/EDS).

## 2. Materials and Methods

### 2.1. Biomaterials Preparation

The Mg-doped ZrO_2_ bioceramics, i.e., Zr_1−x_Mg_x_O_2_ (x = 0.05, 0.1, 0.15, 0.2, 0.25 and 0.3), were prepared by solid-state reaction at high temperature using the procedure as previously reported [20,21,22]. High-purity ZrO_2_ (Riedel-de Haën AG, Seelze, Germany, 99%) and MgO (Alfa Aesar, Karlsruhe, Germany, 99.99%) powders were used as raw materials. The particle size analysis of the raw materials, ZrO_2_ and MgO powdered oxides, measured in suspension using a micro- and nanoparticle analyzer SALD-7101 (Shimadzu, Tokyo, Japan), revealed a mean particle size of 23.257 µm (SD = 0.359) for ZrO_2_, whereas the MgO consisted of particles with a mean size of 1.780 µm (SD = 0.853) [22]. Further, the starting powdered oxides were mechanically activated using a laboratory agate mortar and pestle to obtain the experimented compositions. To obtain cylindrical pellets of 1 g and 10 mm in diameter, the powders were mixed with 5% polyvinyl alcohol (PVA) and uniaxially cold pressed using a metallic dye and a pressure of about 0.5 tons, by a Carver Inc., hydraulic press (Carver Inc., Wabash, IN, USA). Afterwards, the obtained pellets were placed in alumina crucibles and sintered in air for a 12 h dwell time at 1600 °C, with a heating and cooling rate of 5 °C/min. Sintering was carried out using an LHT 04/16 High-Temperature Furnace (Nabertherm GmbH, Lilienthal, Germany).

### 2.2. In Vitro Biomaterials Stability

The stability of the prepared bioceramics (pellets) was tested using commercial saliva substitute gel (Xerostom^®^, Biocosmetics Laboratories, Madrid, Spain). Xerostom^®^ is a natural moisturizer helping to manage symptoms associated with dry mouth and Xerostomia seeks to improve quality of life. Xerostom^®^ ingredients are formulated at a neutral pH, have a mild lemon aroma, and include olive oil, betaine, xylitol, fluoride, vitamin E and vitamin B5. The Xerostom^®^ saliva substitute gel contains the following ingredients: glycerin, aqua, xylitol (10%), potassium citrate, betaine, carbomer, tetrapotassium pyrophosphate; ollea Europaea fruit oil (extra virgin olive oil/aceite de oliva virgen extra), calcium lactate, xanthan gum, aroma, potassium phosphate, sodium benzoate, panthenol (provitamin B5), tocopheryl acetate (provitamin E), carum petroselinum seed oil (parsley oil), sodium propylparaben [23,24].

The stability of the samples was assessed under static conditions in substitute saliva gel (Xerostom^®^). The pellets of around 1 g for each composition were immersed in Xerostom^®^ and maintained at 37 °C in a climate chamber (Binder, Germany) for 2 months. Finally, the pellets were washed with ultrapure water and dried at room temperature before XRPD, FTIR and SEM/EDS analysis.

### 2.3. Bulk Density and Apparent Porosity Measurements

The bulk density (BD) and apparent porosity (AP) of sintered ceramics were measured in aqueous media according to the Archimedes principle using Precisa hydrostatic balance (XB 220A, Precisa Instruments Ltd., Dietikon, Switzerland). Initially sintered samples were weighed in a dry state and immersed in water, where they were kept boiling for 2 h to ensure that water completely filled the open pores. Then, the suspended weights were calculated. For the experiments in this study, 3 sintered samples from each composition were measured and the arithmetic mean was calculated. Further, the apparent porosity and bulk density of different compositions were calculated using the relations:

AP = [(w_3_ − w_1_)/(w_3_ − w_2_)] × 100 (1)

BD = w_1_/(w_3_ − w_2_) (2)

where: w_1_—the mass of the dry sample (in the air), w_2_—the mass of the immersed sample (in the distilled water) and w_3_—the mass of the wet specimen after removal from water.

### 2.4. Structural and Morphological Analysis

Structural and morphological characterization of the prepared biomaterials, pre- and post-immersion, was accomplished by X-ray powder diffraction (XRPD), Fourier transform infrared spectroscopy (FTIR) and scanning electron microscopy/energy-dispersive spectroscopy (SEM/EDS) analysis.

An XRPD analysis was performed to investigate the structure of the samples using a Shimadzu XRD-6000 diffractometer operating at 40 kV, 30 mA, with a Ni- filter and graphite monochromator for CuKα (λ = 1.54060 Å). The diffraction patterns were recorded in the 2θ range of 10–80° at a scan speed of 2 °/min.

Fourier transform infrared (FTIR) absorption spectra were acquired using a Jasco 6200 spectrometer (Jasco, Tokyo, Japan) with Spectra Manager software. The measured samples were in the form of KBr pellets, measured in the range of 400 and 4000 cm^−1^, with a spectral resolution of 4 cm^−1^ and 256 scans. The pellets were obtained by pressing with 5 tons a solid mixture of approximately 2 mg of sample and 200 mg of KBr, placed in a special matrix of approximately 11 mm diameter. A background spectrum of the KBr pellets was recorded under the same instrumental conditions and automatically subtracted from each sample spectrum. Data analysis was performed using Spectra Analysis software.

SEM/EDS images were obtained at 30 kV, 10 μA, with different magnifications, using a Hitachi SU8230 (Tokyo, Japan) microscope. The electron microscope was coupled with an Aztec X-Max 1160 EDX detector (Oxford Instruments, Abingdon, UK). For sample preparation, the material was fixed with double-sided carbon tape and grounded with silver paste, then sputter-coated with 10 nm of gold in an Argon environment.

## 3. Results and Discussion

### 3.1. Bulk Density and Apparent Porosity

The bulk density and apparent porosity of the zirconia-doped samples with different amounts of MgO, which were sintered at 1600 °C for 12 h, are shown in Table 1. The composition of the lowest amount of MgO, x = 0.05, had the greatest bulk density of 4.48 g/cm^3^. The sample with the amount of magnesia, which was increased by 15%, had a minimum bulk density of 3.03 g/cm^3^. Further, when increasing the amount of MgO in the samples, the bulk density slightly increased, with a density of 3.46 g/cm^3^ for the x = 0.3 composition, which had the highest apparent porosity of 40.06%. By increasing the amount of Mg in the samples, the apparent porosity increased from 22% to 40.06%, while the density decreased from 4.48 to 3.46 g/cm^3^. The results revealed that the porosity of the samples increased by increasing the amount of Mg doping.

Porosity strongly influences the mechanical properties of ceramic materials, as a higher porosity reduces the overall mechanical strength. However, from the perspective of biomedical applications, pore size can influence the osteoconduction process. It is known that a lower volume fraction of porosity and finer pores should be beneficial for biological cell attachment [25]. For the growth of osteoblasts, for example, porous surfaces are critical because cells are attached to the pore and can spread through this interconnection. Therefore, the activity of osteoblast is better on porous surfaces than on simple rough surfaces [26,27]. It was demonstrated that the porosity, pore size and even pore interconnectivity of a ceramic material could affect cell behavior, angiogenesis and bone ingrowth in porous ceramics, but higher porosity could affect their mechanical properties [28,29,30].

Moreover, it was observed that the osseointegration properties of zirconia ceramic were better than titanium, a non-absorbable bone graft material. The studies of Sollazzo et al. and Langhoff et al. revealed that zirconia had specific biological effects and a bone-to-implant contact significantly greater if compared to titanium. Based on their results, the osteoblast cell activity on zirconia was higher than on titanium due to the interconnecting pores of zirconia scaffolds, which enhanced proliferation and cell differentiation [27,31,32].

### 3.2. Structural Characterization 

**Figure 1:**
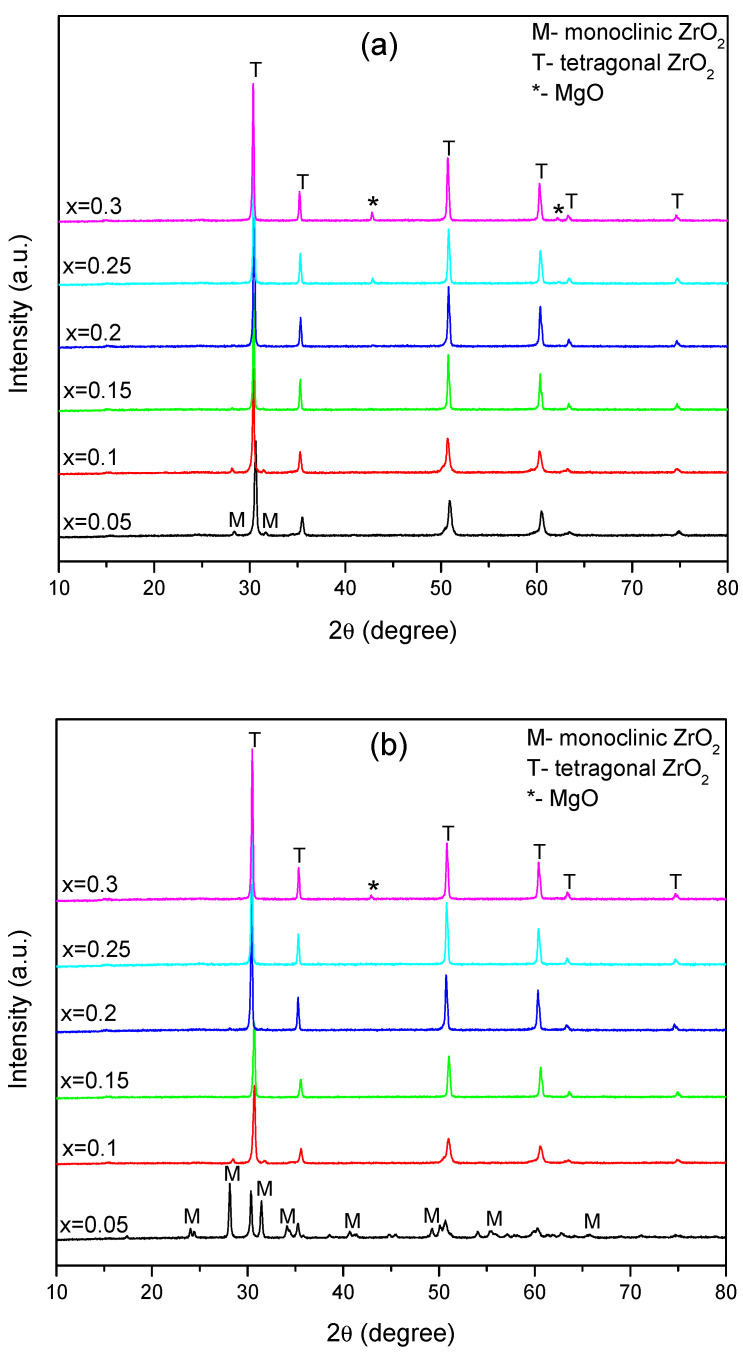
XRPD patterns of compositions belonging to Zr_1−x_Mg_x_O_2_ (x = 0.05, 0.1, 0.15, 0.2, 0.25, 3) bioceramics (**a**) before and (**b**) after immersion in Xerostom^®^.

To determine the structural properties, XRPD analyses were performed both on sintered MgO-doped ZrO_2_, as well as after immersion in Xerostom^®^ saliva substitute gel. The XRPD patterns of the prepared compositions before and after immersion are shown in Figure 1a,b, respectively.

The examination of XRPD patterns pre-immersion of the lowest Mg doping compositions (x = 0.05 and 0.1) revealed polymorphic powders because monoclinic (m-ZrO_2_) and tetragonal (t-ZrO_2_) structure are exhibited. The phase identifications and crystallographic information files corresponding to the pure monoclinic (m-ZrO_2_, PDF # 96-152-8985 [33]) and pure tetragonal (t-ZrO_2_, PDF # 96-230-0613 [34]) phases of ZrO_2_ were selected from the Crystallography Open Database (COD) using Match! software. By increasing the Mg amount on the samples (0.15 ≤ x ≤ 0.3), the powders were mainly composed of t-ZrO_2_, without peaks belonging to the m-ZrO_2_ phases. Moreover, at a higher Mg doping level (x = 0.25 and 0.3), peaks corresponding to MgO (Periclase, PDF#96-900-8672 [35]) were observed, as is visible from Figure 1a. The XRPD analysis showed diffraction peaks at 2θ of 42.86° and 62.37°, which can be assigned to the (200) and (220) planes of MgO. The PDF standard patterns of the t-ZrO_2_, m-ZrO_2_ and MgO are presented together in Figure S1 (Supplementary information file). 

The XRPD patterns post-immersion are presented in Figure 1b. The evaluation of the XRPD patterns revealed the structural stability of the t-ZrO_2_ phase, excepting the x = 0.05 composition, but always accompanied by the m-ZrO_2_ for x = 0.05 and x = 0.1 samples and the MgO secondary phase at a high doping level (x = 0.3).

An important phase transformation took place for x = 0.05 composition after immersion, where important changes in the m-ZrO_2_ and t-ZrO_2_ weight phase ratio occurred. Before immersion in Xerostom^®^ saliva substitute gel, the sample consisted of 90.4 wt.% t-ZrO_2_ and 9.6% wt.% m-ZrO_2_. It was observed that after immersion, an abundance of monoclinic phase (~71.9 wt.%) was induced, as the Xerostom^®^ could remarkably facilitates the t- to m-ZrO_2_ phase transformation from the ceramic sample. After immersion, the sample consisted of 28.1 wt.% t-ZrO_2_ and 71.9 wt.% m-ZrO_2_, respectively. The XRPD patterns of composition x = 0.05 pre- and post-immersion with the diffraction peaks associated with (hkl) planes of tetragonal and monoclinic orientation are revealed in the supplementary information file (Figure S2).

As seen in Figure 1a,b, the XRPD patterns of the fabricated Mg-doped zirconia bioceramics, except the x = 0.05 composition, were almost identical to that after immersion. This can indicate that the synthesized ceramics were not subjected to potential structural degradation during the immersion in Xerostom^®^ saliva substitute gel.

In any case, pre- or post-immersion, no peaks belonging to the MgO phases were observed at a low Mg doping level (0.05 ≤ x ≤ 0.2), confirming the solubility of Mg in the ZrO_2_. Due to the small difference between the ionic radii of the Zr^4+^ (0.84 Å) and Mg^2+^ (0.72 Å), Mg could be easier solubilized in the ZrO_2_ lattice; consequently, the Zr^4+^ on its lattice site was substituted by the Mg^2+^ ion. As stated before, since Mg has an oxidation state of +2, some oxygen vacancy is induced in the structure, which is the main reason for the stabilization of the t-ZrO_2_ [36,37].

The crystallite sizes (D) were calculated using the Scherrer formula [38]:

(3) $$D_{\mathrm{hkl}}=\frac{k\lambda }{\beta \;\cos\theta }$$

where D—crystallite size along (hkl) direction, k—the Scherer’s constant (k = 0.94), λ—wavelength of X-ray, β—full width half maximum (FWHM) of the most intense diffraction line, θ—the Bragg angle.

The evolution of the crystallite sizes in the Mg-doped ZrO_2_ ceramics estimated by Scherrer formula, pre- and post-immersion, is presented in Table 1. The crystallite size varied in the nanometric domain with an average of 36.09 to 55.02 nm.

### 3.3. FTIR Investigations

**Figure 2:**
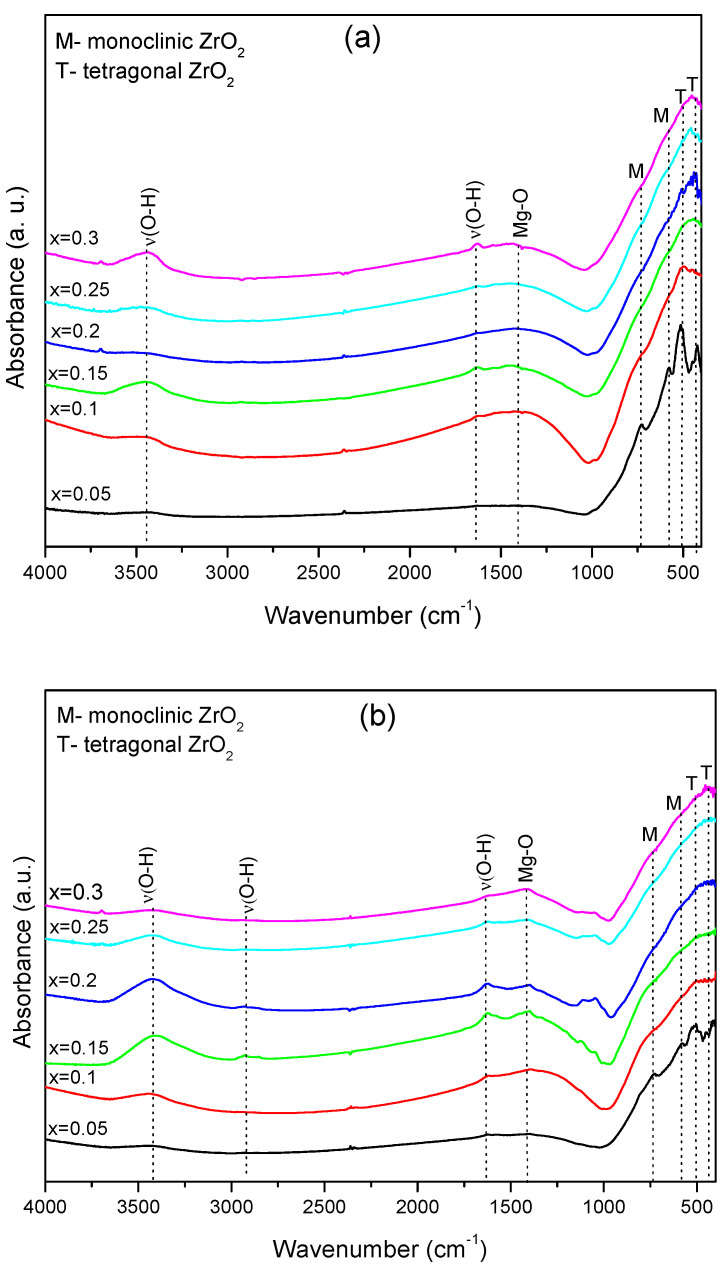
FTIR spectra of compositions belonging to Zr_1−x_Mg_x_O_2_ (x = 0.05, 0.1, 0.15, 0.2, 0.25, 0.3) bioceramics (**a**) before and (**b**) after immersion in Xerostom^®^, in the spectral domain 4000 − 400 cm^−1^.

The FTIR spectra of the investigated samples of Zr_1−x_Mg_x_O_2_ (x = 0.05, 0.1, 0.15, 0.2, 0.25, 0.3), before and after immersion in saliva substitute gel (Xerostom^®^), are shown in Figure 2a,b, and Table 2 reveals the vibration bands for the investigated samples. 

The FTIR analysis of the spectra obtained for the as-prepared samples (Figure 2a) show the presence of two bands observed around 3429 and 1634 cm^−1^ due to the presence of the hydroxide group from water absorption during testing. The band observed at 1440 cm^−1^ can be due to Mg-O interaction [39]. The characteristic bands ascribed to the m-ZrO_2_ are observed at 579 and 722 cm^−1^, and t-ZrO_2_ is 517 cm^−1^ [40,41] (Figure 2a). For the zirconia phases, shifts in wavenumber for the t-ZrO_2_ and m-ZrO_2_ phases have been reported in binary oxides prepared by the addition of divalent or trivalent oxides [42]. The incorporation of these oxides causes lattice deformation on the crystalline structure, with subsequent modification on the force constants of Zr-O and related bonds [43].

The FTIR spectra obtained on the samples after immersion in the Xerostom^®^ show differences in the intensity of the vibration bands corresponding at Zr-O (Figure 2b). Thus, from the comparison of the FTIR spectra of the sample with the composition x = 0.05 pre- (Figure 2a) and post-immersion (Figure 2b), the bands attributed to t-ZrO_2_ decrease in intensity after immersion, and the band attributed at m-ZrO_2_ increases in intensity. These results agree with the XRPD results after immersion in saliva substitute gel, where important phase transformation occurs from t- to m-ZrO_2_.

In conclusion, the weak changes observed in the XRPD patterns and FTIR spectra for the immersed samples, excepting x = 0.05 composition, demonstrate that the structural units involved in Mg-doped ZrO_2_ are quite stable in the Xerostom^®^ environment.

### 3.4. Morphological Characterization

**Figure 3:**
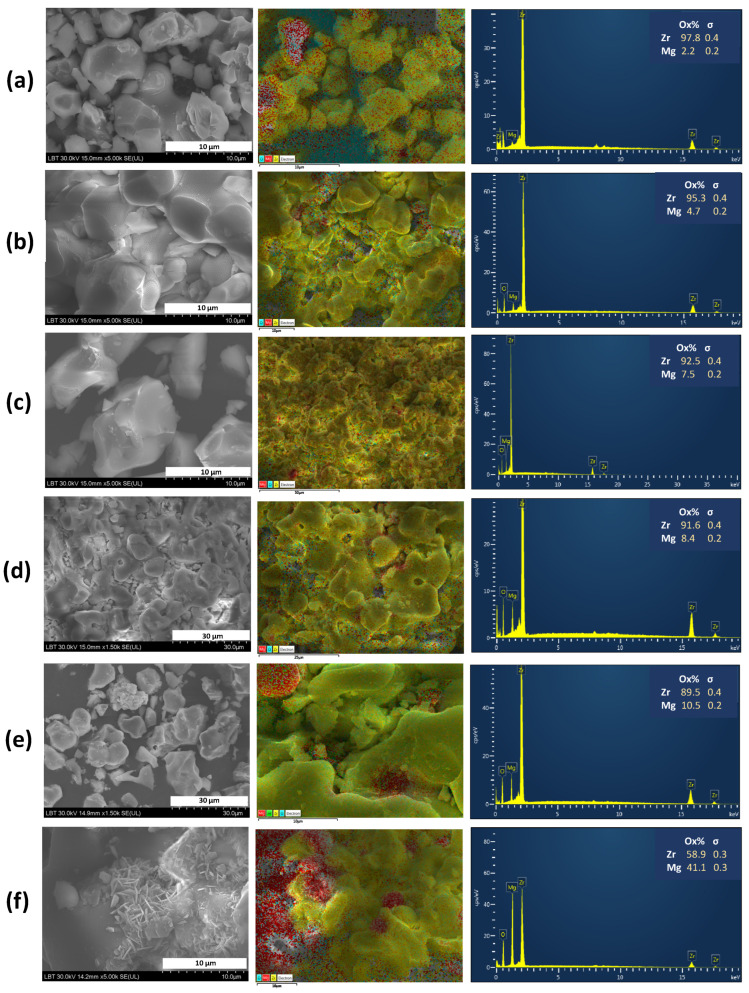
SEM images (**left**), elemental mapping (**middle**) and EDS spectra (**right**) of (**a**) x = 0.05, (**b**) x = 0.1, (**c**) x = 0.15, (**d**) x = 0.2, (**e**) x = 0.25 and (**f**) x = 0.3 compositions belonging to the Zr_1−x_Mg_x_O_2_ system, before immersion in Xerostom^®^ saliva substitute gel. In the elemental mapping images, the assignment of color for each element is the following: yellow for Zr, red for Mg and blue for O, respectively.

The morphological characterization of the prepared Mg-doped ZrO_2_ ceramic material pre- and post-immersion was carried out using SEM. Figure 3 presents SEM images (left), elemental mapping (middle) and the chemical composition analyzed by EDX (right) of the obtained Mg-doped ZrO_2_ ceramic materials before immersion in Xerostom^®^ saliva substitute gel.

The images from Figure 3a–f (left) show grains strongly interconnected to each other with irregular and agglomerated shaped morphology in the ceramic samples. Moreover, uniform 2D rectangular MgO micro-sheets of minimal thickness and irregular shape, stacked in 3D, are observed (Figure 3f, left). A qualitative analysis of the elements present in Mg-doped ZrO_2_ ceramics, evaluated by elemental mapping (Figure 3a–f, middle), confirmed their homogenous distribution in the 0.05 ≤ x ≤ 0.2 compositions. As shown in the images, the MgO is uniformly distributed, indicating that ZrO_2_ is surface modified by MgO. For the x = 0.25 and x = 0.3 samples, an agglomeration behavior was observed. The results of the elemental EDX analyses, displayed in Figure 3a–f (right), confirmed the presence of the magnesium, oxygen and zirconium elements. In addition, no other signals were detected, indicating the purity of the materials. The EDS results further confirmed the increasing tendency of MgO with varying molar Mg/Zr ratios on the samples, in agreement with SEM and nominal compositions.

**Figure 4:**
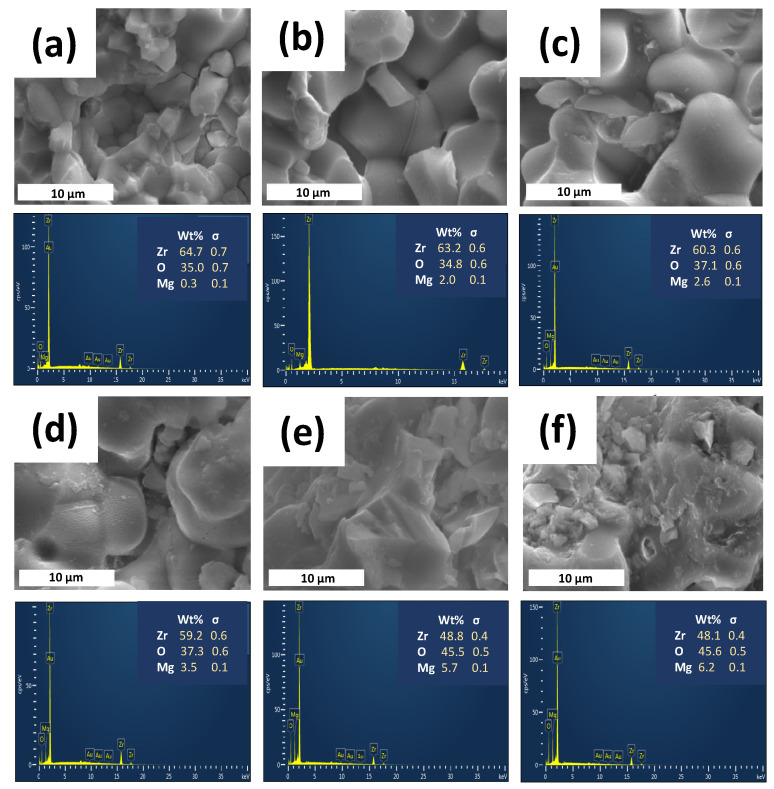
SEM images (**up**) and the corresponding EDS spectra (**down**) of (**a**) x = 0.05, (**b**) x = 0.1, (**c**) x = 0.15, (**d**) x = 0.2, (**e**) x = 0.25 and (**f**) x = 0.3 compositions belonging to the Zr_1−x_Mg_x_O_2_ system, after immersion in Xerostom^®^ saliva substitute gel.

The SEM images after immersion (Figure 4a–f) show that the strong interconnection of the grains was maintained.

This kind of typical interconnected structure, previously observed for YSZ ceramics or ZrO_2_-based composites, allows high mechanical strength to be obtained [20,44,45]. Additionally, the microstructural observations reveal the presence of pores, as seen in Figure 4. It is highly recognized that cellular responses are highly affected by biomaterial porosity. Consequently, the investigation of this effect is of particular importance for the development of implanted biomaterials that integrate with bone tissue.

The phase transformation from t-ZrO_2_, the metastable polymorph, to m-ZrO_2_, the stable one, explains the zirconia sensitivity to LTD (low-temperature degradation). One major property that affects the lifetime of zirconia is hydrothermal aging. The metastable t-ZrO_2_ at the material surface can transform with water or humidity contact, such as body fluids [46], a phenomenon known as LTD, which was first described by Kobayashi et al. [47]. The mechanism by which moisture catalyzes the transformation from t- to m-zirconia was studied by many authors. The current explanation found in the literature is that moisture, in the form of OH ions, diffuses into the zirconia lattice and fills oxygen vacancies, lowering the vacancy concentration and thereby destabilizing the tetragonal phase [48,49]. There are many scenarios proposed for the LTD of t-ZrO_2_ consisting of the following steps: the chemical adsorption of H_2_O on ZrO_2_ surface; the reaction of H_2_O with O_2_ on the ZrO_2_ surface to form hydroxyl ions OH; the penetration of OH into the inner part by grain boundary diffusion; the filling of oxygen vacancies within the grains by OH ions, and thus, the formation of proton defects; and the occurrence of a t–m transformation when the oxygen vacancy concentration is reduced to the extent that the tetragonal phase is no longer stable [48,49,50,51]. A possible explanation of our results can be the following: The main reason for the stabilization of the t-ZrO_2_ is represented by the oxygen vacancies induced in the structure due to the +2 oxidation state of Mg, but the oxygen vacancy concentration is reduced in the x = 0.05 composition, so the t- to m-transformation more easily takes place in the Xerostom^®^ moisture. In contrast, the other samples did not undergo phase transformation, exhibiting stability in the Xerostom^®^ environment.

## 4. Conclusions

Mg-doped zirconia bioceramics in the Zr_2-x_Mg_x_O_2_ (x = 0.05, 0.1, 0.15, 0.2, 0.25 and 0.3) system were synthesized by a facile conventional ceramic method at 1600 °C, followed by their structural, morphological and in vitro characterization by using Xerostom^®^ saliva substitute gel, one of the most common topical dry mouth products used in dentistry. The structural studies pre- and post-immersion revealed t- to m-ZrO_2_ phase transformations during the degradation process in Xerostom^®^, especially at a low Mg doping level (x = 0.05). However, the other studied compositions showed good stability in the Xerostom^®^ environment. A variable apparent porosity of ~30 ÷ 40% was found, which could be beneficial for biological cell attachment. Moreover, the strong interconnection of the grains, which could allow high mechanical strength to be obtained, was maintained after immersion, as revealed by the SEM images. Based on the obtained results of this study, further work is planned to quantify the magnesium and/or zirconium ion release in Xerostom^®^ saliva substitute gel. In addition, further in vivo studies will be carried out to establish the feasibility of using these bioceramics for dental applications.

## Figures and Tables

**Table 1:** The prepared compositions of the Zr_1−x_Mg_x_O_2_ system, samples formula, bulk density, apparent porosity, standard deviation (SD) and crystallite size pre- and post-immersion.

Composition	Formula	Apparent Porosity (%)	SD	Bulk Density (g/cm^3^)	SD	D_Scherrer_ * (nm)	
						Pre- immersion	Post- immersion
x = 0.05	Zr_0.95_Mg_0.05_O_2_	22.00	0.032	4.48	0.122	39.76	45.44
x = 0.1	Zr_0.9_Mg_0.1_O_2_	33.83	0.030	3.61	0.271	48.65	36.09
x = 0.15	Zr_0.85_Mg_0.15_O_2_	32.74	0.037	3.60	0.098	55.02	41.64
x = 0.2	Zr_0.80_Mg_0.2_O_2_	32.79	0.037	3.03	0.343	49.10	52.63
x = 0.25	Zr_0.75_Mg_0.25_O_2_	37.64	0.003	3.31	0.261	53.54	54.84
x = 0.3	Zr_0.7_Mg_0.3_O_2_	40.06	0.036	3.46	0.190	53.29	51.95

* The crystallite size was calculated from XRPD spectra considering the (111) reflection peak of t-ZrO_2_; for the x = 0.05 composition post-immersion, where the m-ZrO_2_ is the predominant phase, the (11$\bar{1}$) and (111) reflection peaks were considered.

**Table 2:** The vibration bands for the investigated samples before and after immersion in Xerostom^®^.

Composition	Pre-Immersion	Post-Immersion
x = 0.05	3458, 1627, 1430 727, 577, 514, 526, 503, 449, 422	3451, 1627, 1573, 1398 725, 580, 526, 517, 503, 462, 452, 421, 414, 407
x = 0.1	3500, 1621, 1428, 499, 454	3451, 1619, 1397, 504, 459
x = 0.15	3453, 2927, 1629, 1441, 1375, 461, 445	3418, 2925, 1620, 1451, 1398, 1127, 1049, 466, 447
x = 0.2	3696, 2922, 1629, 1428, 508, 470, 441, 430, 412	3418, 2930, 1625, 1398, 1107, 1048, 505, 476, 442, 427, 417, 410
x = 0.25	3478, 1629, 1453, 457, 442, 421	3433, 1625, 1418, 1049, 484, 460, 442, 434, 426, 416
x = 0.3	3697, 3440, 1629, 1544, 457,	3697, 3414, 1629, 1422, 1161, 1050, 452

## Data Availability

Not applicable.

## Supplementary Materials

The following supporting information can be downloaded at: https://www.mdpi.com/article/10.3390/ma16072680/s1, Figure S1: PDF standard patterns of the t-ZrO_2_, m-ZrO_2_ and MgO, selected from the Crystallography Open Database (COD) using Match! software.; Figure S2: XRPD patterns of composition x = 0.05 (black, down) pre- and (red, up) post-immersion in Xerostom^®^. The diffraction peaks are associated with (hkl) planes having tetragonal orientation (black, PDF # 96-230-0613) and monoclinic orientation (red, PDF # 96-152-8985), respectively. The crystallographic information corresponding to the t- and m-ZrO_2_ phases were selected from the Crystallography Open Database (COD) using Match! software.
