# Supplementary material for: In Vitro Degradation of Mg-Doped ZrO2 Bioceramics at the Interface with Xerostom® Saliva Substitute Gel
Source: Materials (Basel). 2023 Mar 28;16(7):2680. doi: 10.3390/ma16072680 (PMC10096315; doi:10.3390/ma16072680)
Supplement: Supplementary file 1 [file materials-16-02680-s001.zip › materials-2299150-supplementary.pdf]

Supplementary information

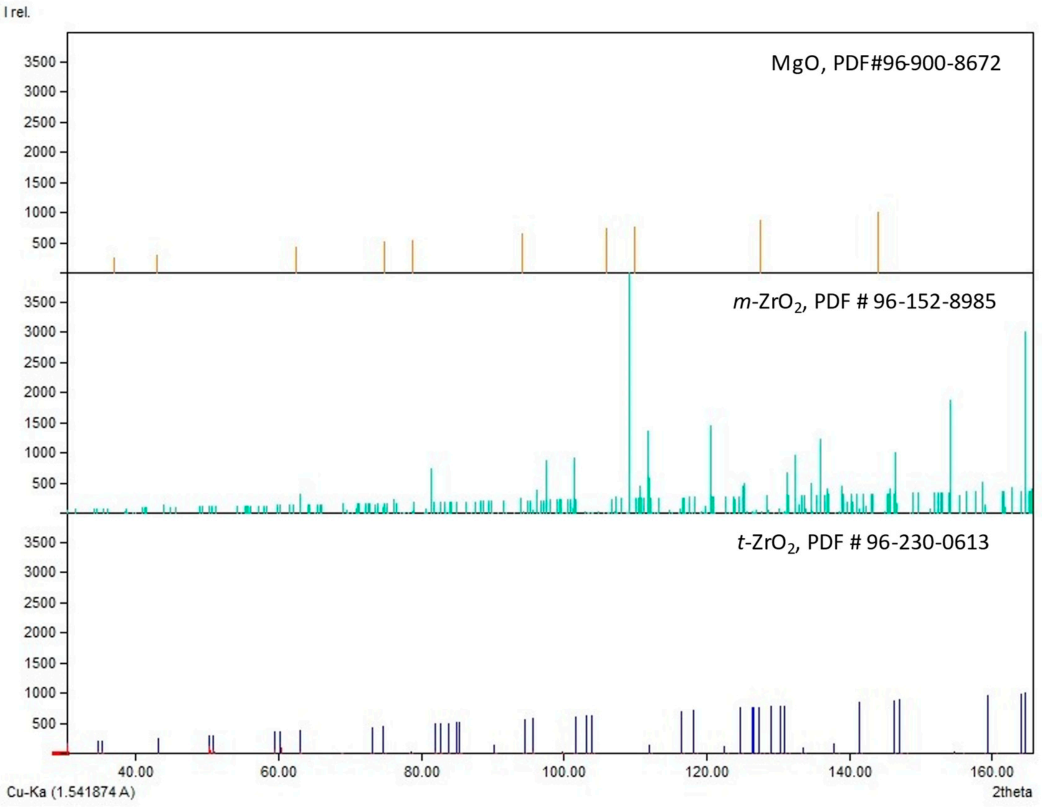

Figure S1. PDF standard patterns of the *t*-ZrO_2, *m*-ZrO_2 and MgO, selected from the Crystallography Open Database (COD) using Match! software.

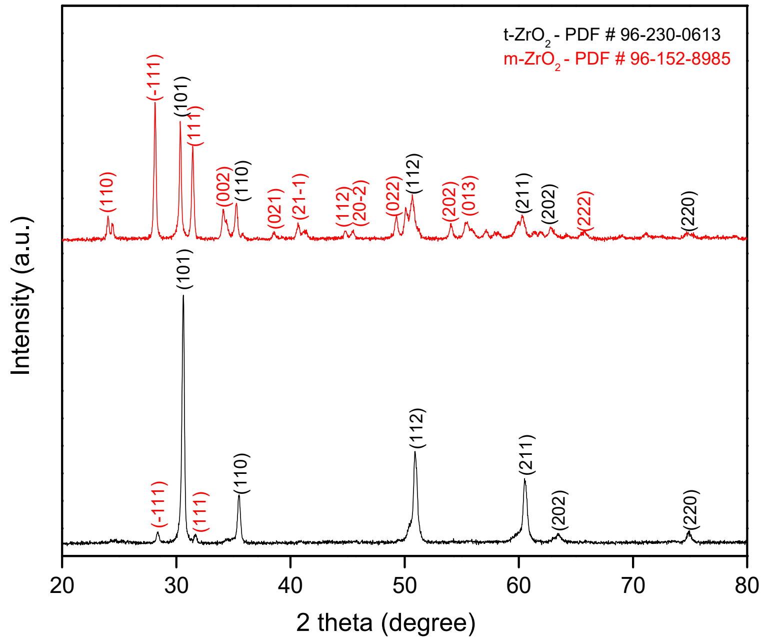

Figure S2. XRPD patterns of composition x = 0.05 (black, down) pre- and (red, up) post-immersion in Xerostom®. The diffraction peaks are associated with (hkl) planes having tetragonal orientation (black, PDF # 96-230-0613) and monoclinic orientation (red, PDF # 96-152-8985), respectively. The crystallographic information corresponding to the *t*- and *m*-ZrO_2 phases were selected from the Crystallography Open Database (COD) using Match! software.
